# Current situation of osteogenesis imperfecta in Spain: results from a Delphi study

**DOI:** 10.1186/s13023-024-03248-0

**Published:** 2024-06-18

**Authors:** Belén Sagastizabal, Inmaculada Calvo, Àngels Martínez-Ferrer, James Clancy, Álvaro Pérez, Alicia Gil, Rosa Bou

**Affiliations:** 1 Department of Paediatrics, Hospital Universitario de Getafe, Madrid, Spain; 2 Paediatric Rheumatology Unit, Hospital Universitario y Politécnico La Fe, Valencia, Spain; 3 Rheumatology Service, Hospital Universitario Doctor Peset, Valencia, Spain; 4 Mereo Biopharma, London, UK; 5 Omakase Consulting S.L, Entença, 332-334, 4º, 1ª, 08029, Barcelona, Spain; 6 Paediatric Rheumatology Unit, Hospital Sant Joan de Déu, Barcelona, Spain

**Keywords:** Osteogenesis imperfecta, Brittle bone disease, Rare disease, Epidemiology, Burden of disease, Clinical management, Unmet needs, Healthcare resource planning

## Abstract

**Background:**

Osteogenesis imperfecta (OI) is a rare disease characterized by low bone mass and bone fragility, associated with an increased risk of fractures, and skeletal and extra-skeletal symptoms that results in an impairment of health-related quality of life of OI patients. Since published studies on OI in Spain are limited, this study aimed to determine the epidemiology, assessed the disease burden, management and unmet needs of OI patients in Spain. Thirty-four experts in the management of patients with osteogenesis imperfecta completed two rounds of online consultation and reported real-life experience and data from Spanish hospitals. Delphi study questionnaires were based on literature review. A working group of nationally recognized clinical experts supported the development of the study questionnaires and the final validation of results.

**Results:**

The estimated prevalence of patients diagnosed with OI in Spain is 0.56:10,000 inhabitants (95%CI: 0.54–0.59), which represents that, approximately, 2,669 OI patients are currently managed in Spanish hospitals. It is estimated that approximately 269 new patients would be diagnosed with OI each year in Spain, representing an estimated incidence of 0.06 (95%CI: 0.05–0.06) per 10,000 inhabitants per year. Clinical management of OI in Spain is performed by a range of medical specialists; however, multidisciplinary care is not fully implemented. The absence of an approved curative treatment or a treatment to reduce the clinical features of the disease remains the main unmet need.

**Conclusions:**

This study provides a snapshot of the current situation of patients with OI in Spain reported by clinical experts. The results provide an estimation of the epidemiology of the disease, and complement the available evidence on disease burden, clinical management, and unmet needs of these patients in Spain.

## Background

Osteogenesis imperfecta (OI) is a phenotypically and molecularly heterogeneous group of inherited connective tissue genetic disorders. OI is associated with low bone mass and bone fragility, which results in an increased risk of fractures and bone deformities [1, 2]. The recurrence of fractures and the comorbidities associated with the disease have a significant impact on patient’s quality of life [3, 4]. People with OI experience different degrees of severity, from milder forms with almost no fractures, in which the diagnosis may go unnoticed, to moderate and severe forms with multiple fractures and deformities over the course of their lifetime. The most severe forms can present intrauterine fractures and death in the perinatal period [5].

OI is a rare disease with an incidence of approximately 1–2 per 10,000 live births [6–8]. Its prevalence varies across studies ranging from 0.5 to 1 case per 10,000 inhabitants in the US and EU [6, 7, 9–11]. In Spain, only a few studies assessing the epidemiology of OI are available [12, 13]. While the Spanish association of brittle bone disease (Asociación Nacional Huesos de Cristal España, AHUCE) estimated that there could be a minimum of 2,700 patients with OI in Spain [14], the Spanish Ministry of Health publishes an annual report about the situation of rare diseases in Spain, in which OI is included. In the latest report from 2022, 13 of the 17 regions estimated a total of 1,194 patients with OI in Spain [15]. The incidence of OI has been estimated at 0.564 per 10,000 in-patients in a hospital study, and at 1.014 per 10,000 new-borns for birth incidence [12].

Curative treatments for OI are not currently available. Clinical management is based on the treatment of signs and symptoms, in which a multidisciplinary group of specialists should ideally be involved [5, 11, 16, 17]. The pillars of musculoskeletal management of OI are physiotherapy and rehabilitation, surgical intervention, and medical treatment [2, 5, 18, 19]. Guidelines for the management of OI patients are available in Spain. However, while specific guidelines are available for paediatric patients, guidelines for adult patients are primarily focused on the management of other, more common bone diseases, such as osteoporosis [5, 20, 21].

In Spain, there are no pharmacological treatments available with an OI indication and no pharmacological therapies have been authorised at EU level. However, some are used off-label in clinical practice [22–24]. Bisphosphonates are antiresorptive drugs widely used to treat OI patients [1, 24]. It has been demonstrated that bisphosphonates improve bone mineral density (BMD) and decrease biochemical markers of bone turnover [16, 24]. In contrast, their effect on fracture reduction is uncertain and in other, non-fracture-related impacts of OI, especially in adults [23–25]. In addition, long-term treatment with bisphosphonates can oversuppress bone remodelling, leading to accumulation of microdamage, which compromises bone mechanical properties and predisposes the patient to fractures [26].

The Delphi methodology is a qualitative research approach that aims to obtain expert view and experience on a real-world problem [27]. In the healthcare setting, it is recommended to study clinical situations for which definitive evidence is scarce [28]. This technique uses a series of consultations rounds to provide information on group opinion and experience. It is characterised by the iteration with experts through a controlled feedback of group opinion [29].

OI is a rare disease that can severely affect patients, and which has limited treatment options and no authorised therapies. Current clinical management is not well defined, and the evidence available about the epidemiology of the condition is limited. This study aimed to ascertain the epidemiology of OI and to obtain a snapshot of the current situation of OI patients in Spain regarding the burden of the disease, its clinical management, and unmet needs, based on the opinion and data reported directly by Spanish physicians with experience in the management of these patients.

## Materials and methods

### Literature review

A literature review focused on OI in Spain was performed during June 2022 to identify publications and data relevant for the development of the Delphi questionnaires. All publications available at that time were included and no time span limits were applied in the search strategy. Inclusion criteria were defined according to the research questions of the Delphi questionnaire. Publications were excluded if they were duplicated, related to animal studies, or mentioned OI but focused on other diseases. The search was conducted using international databases (PubMed) and the Spanish national biomedical database (MEDES). Google searches and the websites of relevant Spanish Scientific Societies and Patient Organisations were used as grey literature sources.

## Delphi study design

The study was conducted using the modified Delphi technique, including a semi-structured, two-round online consultation [30]. The first-round questionnaire was developed based on the results from the literature review. The questionnaire was validated by a Clinical Expert Working Group (CEWG) comprised of three paediatricians and one rheumatologist with recognised experience in the management of OI patients. The first round of consultation with the Delphi panel included 62 questions and was held in October 2022.

The second-round questionnaire was developed based on the analysis of the results from the first round. The objective of the second round of consultation was to obtain a greater degree of alignment in the responses that had shown higher divergence in the first round. A coefficient of variation (CV) smaller than 1 was used to determine agreement in the quantitative questions. Agreement in the qualitative questions was considered when at least 80% of experts agreed on the response option. The questions that did not reach these criteria were reformulated and included in the second-round questionnaire which was validated with the CEWG. The second round of consultation included 22 questions and was conducted in February 2023.

Final study results were presented and discussed with the CEWG in a meeting conducted in March 2023.

## Expert panel

**Fig. 1:**
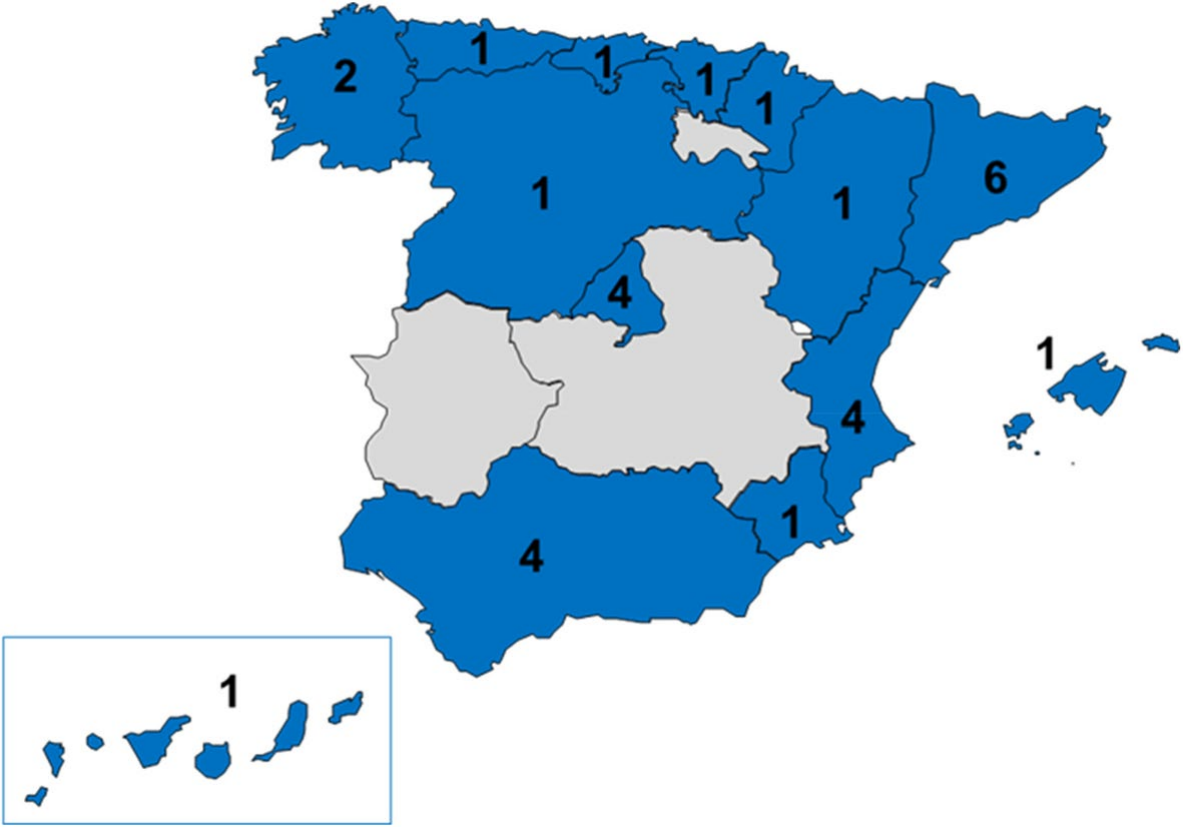
Geographical distribution of Delphi study participants. Footnote: Number of hospitals included in the study per region

Clinicians with experience in the management of patients with OI (defined as having at least 5 patients under their care) were invited to participate in the study. Experts included representation from the medical specialties more frequently involved in the management of OI (i.e. paediatrics, endocrinology, traumatology, rheumatology, internal medicine, genetics). Thirty-four experts, including the four CEWG members, completed the two rounds of consultation. Twenty-nine different hospitals are represented and distributed over 14 out of the 17 Spanish regions (Extremadura, Castilla-La Mancha and La Rioja were not represented). Regional distribution of hospitals that participated in the Delphi study is presented in Fig. 1. Nineteen experts managed primarily paediatrics patients and 13 experts managed mainly adult patients. Two experts were responsible for the management of both adult and paediatric patients and completed the questionnaire for both patient populations separately. No more than one expert from each speciality (paediatric or adult) and hospital was included in the panel. For that reason, 34 experts participated in the study but a total of 36 answers were obtained.

## Statistical analysis

Descriptive statistics were used for data analysis. Quantitative results were analysed calculating the median, first and third quartile, minimum, and maximum values. These are the most appropriate parameters for samples that do not follow a normal distribution [31]. Qualitive questions were analysed as the percentage of experts that selected each option. The results obtained from the questionnaires were analysed both as a total group, and separately for the panel of experts managing paediatric patients (<18 years-old) and for experts managing adult patients (≥18 years-old).

Epidemiology was estimated from the data provided by study participants on the total number of patients already diagnosed and the perceived incidence of newly diagnosed patients in the last 12 months in their hospital. Estimates of prevalence and yearly incidence of each hospital were calculated based on the reported number of patients of each hospital and the population covered by the healthcare area of each hospital. Patients managed in the hospitals but coming from other healthcare areas and regions have not been considered for the epidemiology estimation. Hospital-level prevalence and incidence rates were then extrapolated to regional level, accounting for the population of each respective region. Subsequently, using data collected from participating regions, the total number of OI patients and new cases per year in Spain were calculated by extrapolating regional epidemiological data to the entire Spanish population. The extrapolation was based on the most recent data available from the Spanish National Institute of Statistics (Instituto Nacional de Estadística, INE) [32]. Epidemiology figures are presented per 10,000 inhabitants. Specific paediatric and adult epidemiological estimates are presented per 10,000 inhabitants <18 years-old or ≥18 years-old, respectively. Final epidemiological estimations were validated by the CEWG.

## Results

### Literature review

**Fig. 2:**
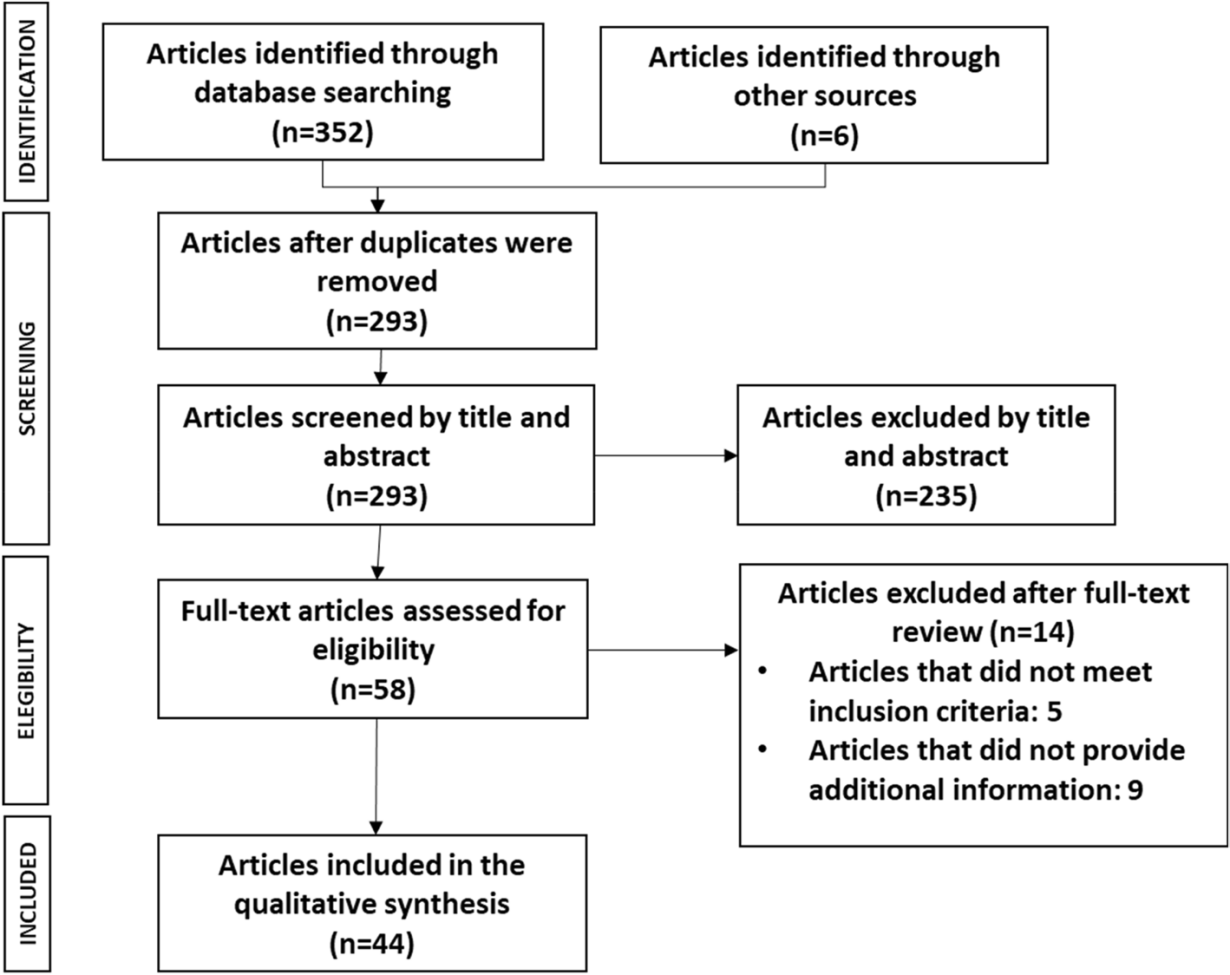
PRISMA diagram for the presentation of the literature review results

A total of 358 publications were retrieved from the searches in biomedical databases (*n* = 352) and grey literature (*n* = 6) sources. A total of 65 duplicated articles were removed. The remaining 293 articles were screened by title and abstract, resulting in the exclusion of 235 articles that did not meet inclusion criteria. The 58 remaining articles were analysed in a full-text review, resulting in the removal of 14 publications. Finally, a total of 44 articles were included in the final evidence synthesis. Figure 2 illustrates the PRISMA diagram summarising the results of the literature review.

## Delphi results

### Epidemiology

Approximately, a total of 2,669 patients (95%CI: 2,570–2,772) would be currently diagnosed with OI in Spain, which represents an estimated prevalence of 0.56:10,000 inhabitants. Among them, 715 (95%CI: 664–769) are children and 1,954 (95%CI: 1,869–2,043) are adults, representing a prevalence of 0.88 (95%CI: 0.81–0.94) and 0.50 (95%CI: 0.48–0.52) per 10,000 inhabitants, respectively. It was estimated that approximately 269 (95%CI: 238–303) new patients are diagnosed with OI every year in Spain which represents an estimated annual incidence of 0.06 (95%CI: 0.05–0.06) per 10,000 inhabitants per year. Approximately, 97 (95%CI: 79–118) would be paediatric and 172 (95%CI: 148–200) adult patients, which represents a paediatric incidence of 0.12 (95%CI: 0.10–0.14) and an adult incidence of 0.04 (95%CI: 0.04–0.05) per 10,000 inhabitants per year. The estimated prevalence and annual incidence of OI in Spain are presented in Table 1.

**Table 1:** Estimated prevalence and annual incidence of OI in Spain

Patient Population	Estimated prevalence	N of patients with OI	Estimated annual incidence	N of newly diagnosed patients with OI annually
Patients with OI	0.56:10,000 inhabitants (95%CI: 0.54 – 0.59)	2,669 (95%CI: 2,570–2,772)	0.06:10,000 inhabitants (95%CI: 0.05 – 0.06)	269 (95%CI: 238–303)
Paediatric patients with OI	0.88:10,000 inhabitants < 18 years-old (95%CI: 0.81 – 0.94)	715 (95%CI: 664 – 769)	0.12:10,000 inhabitants < 18 years-old (95%CI: 0.10 – 0.14)	97 (95%CI: 79 – 118)
Adult patients with OI	0.50:10,000 inhabitants ≥ 18 years-old (95%CI: 0.48 – 0.52)	1,954 (95%CI: 1,869 – 2,043)	0.04:10,000 inhabitants ≥ 18 years-old (95%CI: 0.04– 0.05)	172 (95%CI: 148 – 200)

*Abbreviations*: *N* Number, *OI* Osteogenesis imperfecta, *CI* Confidence Interval

Patients with OI are geographically disseminated, usually needing to travel to other healthcare areas or regions to be diagnosed and followed up. More than half of the patients with OI (53%) under the care of the clinicians participating in the study are referred from other healthcare areas (45%) or other regions (8%) to be diagnosed. Furthermore, 52% of patients are usually referred from other healthcare areas (38%) or from other regions (14%) for their regular follow up.

### Burden of the disease

OI's skeletal and extra-skeletal characteristics and symptoms, including long bone deformities, scoliosis/kyphosis, low BMD, chronic pain, fatigue, and psychological disorders, are more common in adult patients than in paediatric patients, according to the data reported by the participants. Dentinogenesis imperfecta is more frequently diagnosed in paediatric patients because adults often have a worse dentin status naturally, leading to underdiagnosis in adult patients with OI. The median percentage of patients suffering from each skeletal and extra-skeletal characteristic and symptom derived from OI that reached agreement among experts (CV ≥1) are presented in Table 2, along with the interquartile range (IQR) reflecting the variability in the results inherent to the condition.

**Table 2:** Patients suffering from skeletal and extra-skeletal characteristics and symptoms of OI

Characteristic	Whole population	Paediatric patients	Adult patients
Skeletal characteristics			
Long bone deformity	25.0% (IQR 10.0%—30.9%)	20.5% (IQR 8.5%—30.0%)	30.0% (IQR 15.0%—60.0%)
Scoliosis/kyphosis	30.5% (IQR 20.0%—50.0%)	25.0% (IQR 20.0%—32.0%)	50.0% (IQR 35.0%—60.0%)
Low bone mineral density	68.0% (IQR 50.0%—90.0%)	60.0% (IQR 45.8%—82.5%)	82.0% (IQR 60.0%—90.0%)
Extra-skeletal characteristics			
Dentinogenesis imperfecta	27.0% (IQR 15.0%—42.0%)	28.5% (IQR 15.0%—41.5%)	20.0% (IQR 10.0%—54.5%)
Symptoms			
Chronic pain	36.4% (IQR 10.0%—60.0%)	16.4% (IQR 4.8%—50.0%)	50.0% (IQR 30.0%—60.0%)
Fatigue	25.0% (IQR 5.0%—50.0%)	17.5% (IQR 0.0%—43.1%)	30.0% (IQR 10.0%—60.0%)
Psychological disorders	20.0% (IQR 1.2%—30.0%)	15.0% (IQR 0.0%—25.0%)	25.0% (IQR 10.0%—37.5%)

*Abbreviations*: *IQR* Interquartile range

Despite the agreement that clinical features of OI can seriously impact the quality of life (QoL) of patients, most clinicians (89%) do not perform a QoL assessment on a regular basis mainly due to the lack of validated tools and available time in clinical practice.

Experts reported that approximately 22.5% (IQR 10.0%—30.0%) of OI patients suffer at least one fracture per year. Regarding the number of fractures per patient and year, OI patients experience a median of 1 (IQR 0.4–1.2) fracture per year. Considering the type of fractures, 20.0% (IQR 3.4%—29.5%) would be vertebral and 80% non-vertebral. Of these, 31.2% (IQR 10.0%—50.0%) can be classified as milder, 42.6% (IQR 30.0%—54.5%) as moderately severe and 26.3% (IQR 10.0%—40.0%) as severe. For the purposes of the Delphi study, non-vertebral mild, moderate, and severe fractures are defined as fractures not requiring patients visiting a healthcare centre, those requiring medical intervention in a healthcare centre and fractures that require surgical intervention, respectively. Table 3 shows the incidence of fractures in OI patients.

**Table 3:** Incidence of fractures in patients with OI

Characteristic	Whole population	Paediatric patients	Adult patients
Patients suffering 1 fracture in last 12 months (%)			
Median (IQR)	22.5 (IQR 10.0—30.0)	28.0 (IQR 20.0—30.0)	10.0 (IQR 6.0—20.0)
Fractures per patient in 12 months (N)			
Median (IQR)	1.0 (IQR 0.4—1.2)	1.0 (IQR 0.3—1.2)	1.0 (IQR 0.5—1.5)
Vertebral fractures (%)			
Median (IQR)	20.0 (IQR 3.4—29.5)	10.0 (IQR 0.0—27.0)	20.0 (IQR 10.0—30.0)
Non-vertebral fractures (%)			
Mild Mean (IQR)	31.2 (IQR 10.0- 50.0)	31.6 (IQR 11.5- 57.5)	30.6 (IQR 5.0- 40.0)
Moderate Mean (IQR)	42.6 (IQR 30.0- 54.5)	40.4 (IQR 25.0- 53.8)	45.4 (IQR 33.3- 54.5)
Severe Mean (IQR)	26.3 (IQR 10.0- 40.0)	28.0 (IQR 10.0- 40.0)	24.0 (IQR 0.0- 45.0)

*Abbreviations*: *IQR* Interquartile range

OI patients go through a median of 1 (IQR 0.1–1.0) hospitalisation per year, and fractures are the main cause of hospitalisation. In each in-patient period, they stay a median of 3 (IQR 2.0–5.0) days in the hospital. Moreover, during their lifetime, OI patients go through a median of 2 (IQR 1.1–4.0) ambulatory surgeries and 1.9 (IQR 1.0–3.0) surgeries requiring hospitalisation.

### Management

**Fig. 3:**
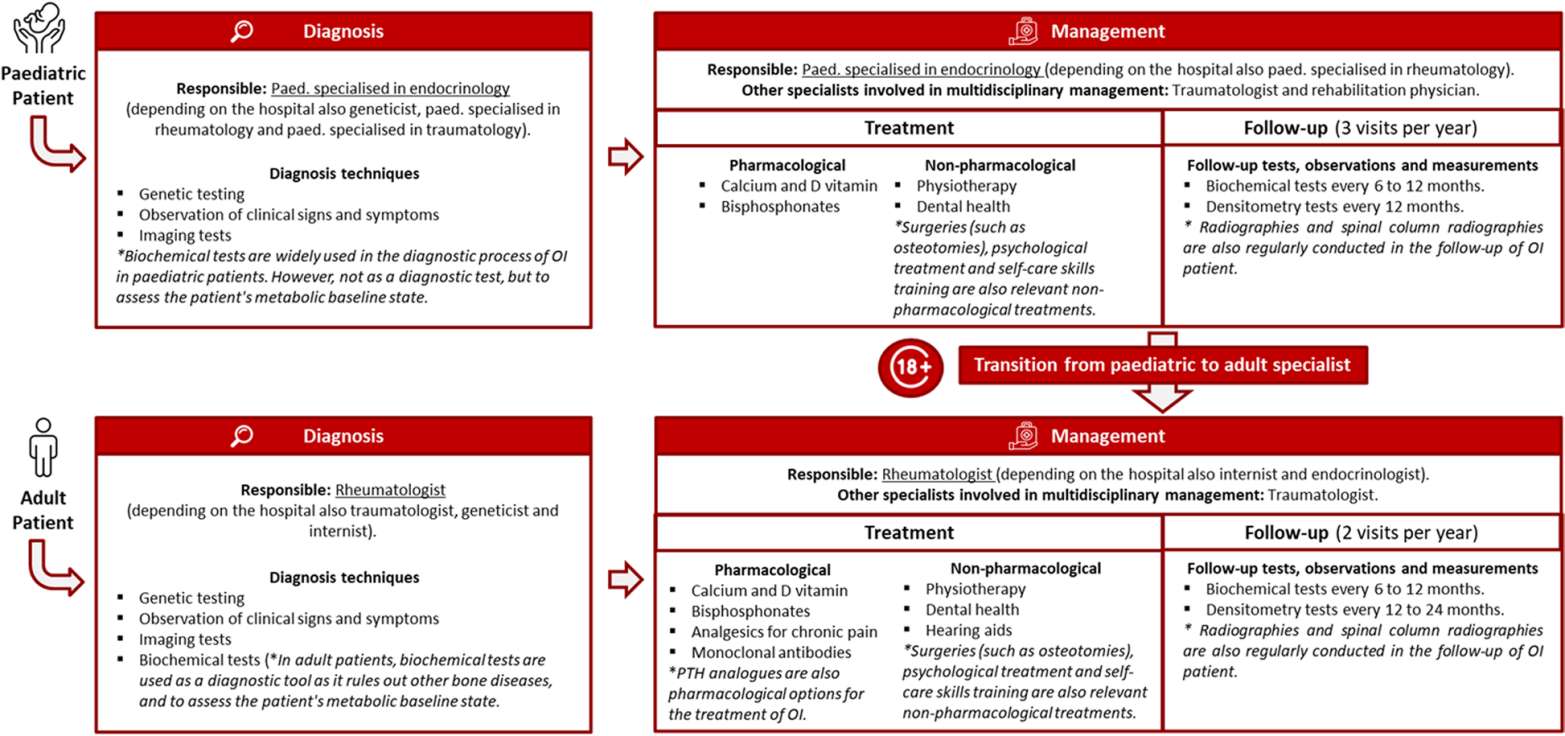
Patient pathway of OI patients in Spain. Footnote: *Qualitative comments made by the CEWG to complete the patient pathway of OI patients in Spain

The clinical pathway followed by paediatric and adult OI patients in Spain has been described by the experts as presented in Fig. 3. The main medical specialties responsible for patient management are paediatrics, endocrinology, rheumatology, traumatology, internal medicine and genetics. The diagnosis and management of paediatric and adult patients, although similar in many aspects, present some differences. Regarding pharmacological treatment, paediatric patients are mainly prescribed calcium and vitamin D supplementation and bisphosphonates. In addition to this, adult patients also receive analgesics for chronic pain and can be prescribed off-label monoclonal antibodies. The most common non-pharmacological treatment was, hearing aids, used to address premature hearing loss due to OI mainly used in adults with OI. Finally, in terms of follow-up, paediatric patients undergo more frequent tests and follow-up visits (3 visits a year), when compared with adults (2 visits a year).

A majority (91%) of experts follow clinical guidelines, consensus documents or specific hospital protocols for OI management. Genetic testing is available in approximately 79% of hospitals and patients are referred to other centres if unavailable. Regarding the key factors driving early diagnosis of OI, the CEWG agreed that these include the awareness of the main signs and symptoms, availability of referral criteria and the coordination between medical specialties. In addition, according to the experts, the median age at diagnosis of OI patients is 3.8 (IQR 1.7–5.0) years-old for paediatric and 17.3 (IQR 6.0–30.0) years-old for adult patients. A median of 40% (IQR 25.0%—90.0%) of the adult patients have been diagnosed during childhood.

According to experts, 70% (IQR 50.0%—81.8%) and 50% (IQR 30.0%—63.0%) of patients are prescribed pharmacological (excluding calcium and vitamin D received by all patients) and non-pharmacological treatments, respectively. The rate of bisphosphonate use is similar in adult (70%, IQR 50.0%—80.0%) and paediatric (75%, IQR 60.0%—100.0%) patients.

Most experts (80%) transition OI patients from the paediatric to the adult specialist at the age of 18. The majority of experts (82%) interact with the paediatric/adult specialist during the transition. Experts considered that the existence of OI units where paediatric and adult specialists can interact is key to optimise patient transition. According to the panel, only 2 hospitals in Spain have officially institutionalised OI units, while some others may have units for the management of bone metabolism pathologies, or multidisciplinary teams which are not officially instituted in the hospital.

### Unmet needs

All participants (100%) considered that there are relevant unmet needs in OI. The panel agreed that the most relevant unmet need is the availability of a treatment that it is either curative or helps with symptom management. Secondly, experts considered multidisciplinary management and referral centres as relevant unmet needs, followed by the availability of consensus documents for the management of OI patients. Table 4 shows the most relevant unmet needs in OI in Spain, prioritised based on the opinion of the experts participating in the study.

**Table 4:** Ranking of most relevant unmet needs of OI in Spain

Ranking	Unmet need
1	Availability of a curative treatment that targets the pathogenesis of OI
2	Availability of an approved treatment targeted at preventing or reducing the clinical features of the disease (e.g. incidence of fractures)
3	Availability of a national consensus document, protocol or guideline for the diagnosis, therapeutic management, and follow-up of OI patients in Spain
4	Multidisciplinary management of OI patients
5	Availability of multidisciplinary reference centres for the management of patients with OI

*Abbreviations*: *OI* Osteogenesis Imperfecta

## Discussion

The management of OI patients varies between centres due to the rarity of OI and limited evidence on standard treatment approaches and outcomes. Despite this, the Delphi panel reached agreement on relevant questions about the current status of the disease in Spain. This study is based on two Delphi consultation rounds. As a result of performing a second round of consultation, participants reached agreement on those questions with higher degree of divergence in the first round. This study presents a summary of the perspective of experts on the epidemiology, burden of the disease, clinical management and unmet needs of OI in Spain.

The estimated prevalence of patients with OI in Spain obtained in this study is 0.56:10,000 inhabitants, which falls within the prevalence range reported in the literature of 0.5 to 1 per 10.000 inhabitants [6, 7, 9–11]. The estimated number of patients of 2,669 is also aligned with the few estimations currently available in Spain, which vary from 1,194 to 2,700 diagnosed patients [14, 15]. Only one national OI patient registry is available in Spain. However, the data from this registry are limited, since not all hospitals within each region report data, and not all regions are included. Therefore, this registry reports a minimal number of diagnosed cases (1,194), and it is estimated that there are more OI patients diagnosed in Spain who are not included in this registry, which supports the results of the study. The prevalence of paediatric and adult patients estimated in the study is 0.88:10,000 inhabitants <18 years-old and 0.50:10,000 inhabitants ≥18 years-old, respectively. The difference observed between the epidemiology of both patient populations is considered representative of clinical practice, considering that paediatric patients are more easily identified and better controlled than adult patients, and hence the diagnosis rate in children is greater [2].

This study estimated an incidence of 0.06:10,000 inhabitants per year. Currently OI incidence is usually reported as birth incidence. A retrospective study of admission records from OI patients in specialized care settings between 2000 and 2017 in Spain indicates an incidence at birth of 1.014 per 10,000 children, meaning 34 new cases would be diagnosed each year in Spain [12]. The incidence data obtained in our study is remarkably greater, since it includes patients diagnosed in Spain at all ages, not only at birth. Both figures, although not comparable, are better understood together and represent current clinical practice, when most patients are diagnosed during their childhood, and only a minority are diagnosed at birth.

Adults present with skeletal and non-skeletal characteristics and symptoms more frequently than children, based on study results. Adult patients are more often lost in follow-up and therefore experience worse disease symptoms management. A particular concern is pain, since it limits patients' activities and negatively impacts their quality of life [33–35]. Furthermore, there is a low level of consensus regarding the management of adult patients with OI, which is reflected in the lack of specific clinical guidelines. An optimal transition of patients from the paediatric to the adult specialist is critical to reduce these differences, and to overall improve patient care and health outcomes for adult patients, including reduction of fractures, skeletal and non-skeletal characteristics, and symptoms of the condition. Inadequate paediatric to adult transition programs and the lack of multidisciplinary transition units where clinicians coordinate patient transition may lead to increased morbidity and discontinuity of care. In order to effectively transition patients with complex or chronic health conditions, programs that take into account their preferences are needed [36].

Study results highlighted that the main unmet need of OI patients in Spain is the availability of a treatment that is either curative or can help with symptom management. Although there is still no medical treatment approved for OI, new treatments are currently under clinical development that could address this unmet need. These future therapeutic options could help reduce fractures, even to a full extent, thus reducing the main cause of disease burden [37–39]. Participants also considered the multidisciplinary management of OI and availability of multidisciplinary referral centres as key unmet needs. Therefore, experts agreed that it is critical to work towards multidisciplinary management with the aim of improving the overall health outcomes of OI patients.

## Limitations

As in other similar studies, the Delphi methodology has proven useful and allowed to obtain information and data based on the opinion and current clinical practice experience of experts in Spain [40–43]. However, the present study is not without limitations, some of which are inherent to the methodology. Clinical practice among different hospitals can be variable, especially considering the lack of clinical guidelines in OI. To address this, the Delphi panel was large, multidisciplinary, and widespread over 29 different hospitals and 14 of the 17 regions of Spain, with the aim of representing, as much as possible, the reality and heterogeneity of clinical practice. Additionally, the inclusion of hospitals with different sizes and levels of expertise in the management of OI may have produced a wider variability of responses than if only reference centres were included. For that reason, two rounds of consultation were performed following the modified Delphi approach, with the second round intended to obtain a greater degree of alignment in those answers showing a higher degree of dispersion during the first round. Finally, our epidemiological results, based on extrapolations, represent the current situation of OI patients in Spain. However, epidemiological studies designed for this purpose must be conducted, along with the establishment of patient registries or databases that can be analysed and provide robust data.

## Conclusion

To our knowledge, this study represents the first attempt to estimate the epidemiology of OI in one country in Europe using Delphi methodology. The obtained results present an estimation of the prevalence and incidence of the condition in Spain and complement the available evidence of the burden of the disease, management, and unmet needs. These findings can also provide additional information for informed decision making for patients, clinicians, and the Spanish national health system. Finally, the study has also highlighted the main areas of patient management that need improvement in upcoming years: the availability of consensus documents and treatment guidelines, the establishment of multidisciplinary management throughout the patient's life, and especially the approval of the new treatments under development specifically for OI that can help patients minimise the burden of disease, primarily by reducing fractures.

## Data Availability

The datasets used and/or analysed during the current study are available from the corresponding author on reasonable request.

## Abbreviations

AHUCE: Asociación Nacional Huesos de Cristal España
BMD: Bone Mineral Density
CEWG: Clinical Expert Working Group
CI: Confidence interval
CV: Coefficient of variation
EU: European Union
INE: Instituto Nacional de Estadística
IQR: Interquartil range
OI: Osteogenesis imperfecta
QoL: Quality of Life
US: United States
